# Would you Bribe your Lecturer? A Quasi-experimental Study on Burnout and Bribery in Higher Education

**DOI:** 10.1007/s11162-021-09669-1

**Published:** 2021-12-02

**Authors:** Kristina S. Weißmüller, Lode De Waele

**Affiliations:** 1 KPM Center for Public Management, Universität Bern, Bern, Switzerland; 2 Department of Organization Studies, Tilburg University, Tilburg, The Netherlands; 3 Research Group on Politics and Public Governance, University of Antwerp, Antwerp, Belgium

**Keywords:** Higher education, Bribery, Burnout, Commitment to public interest

## Abstract

Bribery is a complex and critical issue in higher education (HE), causing severe economic and societal harm. Traditionally, most scholarship on HE corruption has focused on institutional factors in developing countries and insights into the psychological and motivational factors that drive HE bribery on the micro-level mechanisms are virtually non-existent. To close this research gap, this study investigates the connection between study-related burnout and university students’ willingness to offer bribes to their lecturers to pass important exams. Conducting a vignette-based quasi-experimental replication study with 624 university students in Germany, Belgium, and the Netherlands we find that university students in three countries differentiate sharply between different shades of bribery and that a majority accept using emotional influence tactics to pass (failed) exams. In contrast, offering a helping hand or money (i.e., darker shades of bribery) to their lecturer was less acceptable. Study-related burnout is associated with a higher likelihood of engaging in these darker shades of bribery and students’ commitment to the public interest is but a weak factor in preventing unethical behavior. In summary, this study provides solid empirical evidence that university students are likely to use emotional influence tactics violating both the ethical codes of conduct and the formalized bureaucratic procedures of HE examination, particularly if they suffer from study-related burnout. However, the accelerating effect of burnout on bribery is conditional in that it only holds for darker shades of bribery. HE institutions may benefit from implementing the four-eye principle and from launching awareness campaigns that enable lecturers to better recognize these tactics and engage students in creating a transparent environment for testing, grading, and collaboration that is resistant to bribery.

**Supplementary Information:**

The online version contains supplementary material available at 10.1007/s11162-021-09669-1.

## Introduction

Bribery is a substantial and critical issue in institutions of higher education (HE) worldwide (Heyneman, 2014; Heyneman et al., 2008; Johnston, 2001). As a complex phenomenon that causes severe economic and societal harm, bribery is a form of corruption rooted in both psychological, i.e., individual, and circumstantial, i.e., institutional factors. Plainly, bribery describes an individual’s unethical attempt to persuade someone else to make them do something for them by giving that person something that they want. Specifically, Ramdani and van Witteloostuijn (2014) define bribery as “the corrupt payment, receipt, or solicitation of a private favour for actions or decisions from influential or powerful agents or authorities which could be public officials, corporations or people inside corporations to generate private benefits of the briber.” In HE, bribery often involves acts of dishonest, unethical, and socially unaccepted or illegal behavior committed by both students and academic staff (Chapman & Lindner, 2016; Waite & Allen, 2003). Common forms of bribery in HE involve buying personal favors and university degrees (Feoktistova, 2014), undue promotion of faculty staff, and the corrupt management of public funds and property (Osipian, 2007).

Many people associate HE bribery with developing or less-industrialized countries because HE institutions in these countries are particularly challenged with ensuring staff and student compliance (Kremer, 2003) while dealing with unreliable and ineffective bureaucracies, inadequate public funding, and political support leading to insufficient remuneration of teaching staff that may incentivize bribery and unethical behavior (Rumyantseva, 2005). However, this stereotypical picture is incomplete: In fact, bribery in HE institutions is also prevalent in developed and industrialized countries, and it is not a marginal problem either. Heyneman et al. (2008), for instance, show that more than 60% of students in European countries such as Bulgaria, Croatia, Moldova, and Serbia report that bribing lecturers for passing exams is common among their schools. Recently, about fifty affluent U.S. citizens were convicted for bribing university admission pathways originally designed for student athletes to buy access to elite universities for their children (Downes, 2017). A similar case against Harvard University revealed that quid-pro-quo donations by financially strong private donors played a significant role in providing elite university access in the U.S. for students who would not be admissible to these institutions by the legal and ethical standards in place (Glendinning et al., 2019).

Bribery in academia can have dramatic effects for social cohesion, undermining the fundamental principle of equity, and the general public’s trust in institutions (Denisova-Schmidt, 2018; von Arnim, 2003). Although bribery is often described as a *victimless crime*, von Arnim (2003) points out that this is in fact untrue: Even though bribery might not create *one* specific victim—in contrast to other crimes such as robbery or murder—bribery is a crime that will always indirectly harm the welfare of a substantial number of people. Bribing lecturers to pass exams enables people to receive HE degrees without the required intellectual capacities to effectively achieve them. As a result, incompetent and corrupt individuals may gain inappropriate access to powerful political and managerial positions in public and private organizations by bypassing the selection process institutionalized in academia and by abusing the signaling effect of (unduly acquired) academic degrees (Heyneman, 2014; Heyneman et al., 2008; Osipian, 2007). In the long term, bribing for university access or passing exams—for instance—also has substantially negative effect on societal welfare by undermining procedural equity, access equality, and performance quality (Heyneman, 2014; Osipian, 2007) to a degree that bribery in HE even impedes economic growth by relatively slowing down the process of accumulating human capital in those (honest) students left behind because they do not bribe, hence diminishing societal progress, social mobility, innovation, and inhibiting citizen equality (Heyneman, 2014; Osipian, 2007). In sum, HE bribery is a particularly dramatic issue with long-term and high-stake leverage effects (Denisova-Schmidt, 2018; von Arnim, 2003).

Besides institutional factors received most scholarly attention in the past [see, e.g., Johnston (2001), Heyneman (2014), and den Nieuwenboer and Kaptein (2008)], recent studies also link bribery to individual micro-level conditions, that is motivational, psychological, and attitudinal factors nested within the individual. These individual factors, however, by and large remain understudied because bribery—like any unethical and socially undesirable behavior—is hard to measure (Feoktistova, 2014; Osipian, 2007, 2008; Waite & Allen, 2003). The particular lack of empirical research into HE bribery is especially worrying given that recent medial outcry (see US cases mentioned earlier) has shed light onto HE corruption as an alarming signal for the erosion of the ethical standards of objectivity and honesty employed by agents within HE institutions. This is a fundamental issue, because the commitment to ethical standards and the public interest marks the value-based foundation that justify the traditional privileges and the autonomy granted to institutions of HE by the general public (Altbach, 2005; Heyneman, 2014).

To fill this research gap, we turn our attention towards these micro-behavioral (i.e., motivational and psychological) foundations of HE bribery to better understand why some students would attempt to bribe their lecturer while others do not. In this, we respond to prior calls for research (Chapman & Lindner, 2016; Makel & Plucker, 2014; Osipian, 2008; Petrov & Temple, 2004) pointing out the need for more scholarship into the personality aspects influencing likelihood of engaging in acts of bribery in the context of HE.

Within the scope of this study, we are especially interested in studying the effects of two individual attributes on HE students’ likelihood to bribe: burnout and students’ commitment to the public interest (CPI). These attributes gained our attention for three reasons. First, there is ample evidence, connecting burnout with various types of unethical behavior of which bribery is a self-evident example (Pulich & Tourigny, 2004). Second, burnout among students has soared due to the recent Covid-19 pandemic, see, e.g., Boccio et al. (2016), Fernández-Castillo (2021), Jiang (2021), and Stacey et al. (2020). Third, burnout is associated with a general loss of regard for the public interest, and a loss of attachment to the organization and its core values pointing toward a potentially complex relation between burnout, bribery, and CPI (Davis & Welton, 1991; Fritz et al., 1999; Glover et al., 1997; Moore, 2008; Yong & Yue, 2007).

We decided to analyze this theoretical relationship in the context of Western European countries (Germany, Belgium, and the Netherlands) for two reasons. First, bribery among HE institutions in highly industrialized countries remains poorly studied and calls for more empirical exploration. Second, bribery in these countries is typified as illegal and unethical, socially undesirable behavior obstructing empirical research. To account for this issue, this study employs a quasi-experimental research design in which respondents’ intent to bribe was measured with a pre-validated multi-item factorial variable and with systematically varied between-subject randomized vignette treatments. These vignettes reflect different degrees of seriousness of the bribery act to ensure sufficient contextual variance while being set in the typical situation of a one-to-one consultation between a student and a lecturer. Respondents are 624 university students at large public universities in three European countries: Germany, Belgium, and the Netherlands. The quasi-experiment was complemented with a questionnaire on study-related burnout, commitment to the public interest, and attitude-based as well as socio-demographic control variables that are indicative for deviant behavior.

## Conceptual Framework

In this section, we present a conceptual framework against which we derive three hypotheses. The framework is divided into three sections. In the first section, we explain the relation between briber and bribe-taker in the context of different shades of bribe severity. The conceptual framework then turns to the motivational and psychological underpinnings of offering bribes in higher education. In the second section, we explore the relationship between study-related burnout and HE students’ likelihood of engaging in bribery, while the third section concerns the effect of commitment to the public interest on this relationship.

### Bribery in Higher Education

Turow (1985; p. 249) defines bribery as “the act when personal advantage is offered, without the authority of law, to a public official with the intent that public official act favorably to the offeror at any time or fashion in the execution of the public’s official’s duties.” In this, bribery involves “the corrupt payment, receipt, or solicitation of a private favor for actions or decisions from influential or powerful agents or authorities which could be public officials, corporations or people inside corporations to generate private benefits of the briber” (Ramdani & van Witteloostuijn, 2014, p. 1). Consequently, bribery involves two different agents: A briber trying to influence another agent (i.e., the bribe-taker) who has the power to perform a specific action in favor of the briber. In exchange for this action, the briber compensates the bribe-taker with incentives such as financial transfers, discounted access to services or favors, or the prospect of similar reciprocal acts in the future (D'Andrade, 1985). Yet, the briber can also offer emotional stimuli that include the removal of undesirable sentiments such as guilt from the bribe-taker by using soothing and reassuring narratives but also other forms of emotional gratifications (Batson et al., 1995). In the context of HE, the power distance between students and lecturers qualifies the former as potential bribe-offerors and the latter as potential bribe-takers.

As a complex and multifaceted phenomenon, bribery comes in different shades of severity and visibility (Osipian, 2007; Ramdani & van Witteloostuijn, 2014). Heidenheimer (2009) differentiates between *white*, *grey*, and *black* forms of bribery. *Black bribery* is the most direct, transactional form of exchanging money for any type of preferential treatment (i.e., the classic brown envelope in exchange for a favor). *Grey bribery* is also based on a reciprocal exchange between the briber and the receiver of the bribe. However, the bribe-related relationship is based on the exchange of non-monetary goods or services—often with temporal delay. One example could be, for instance, a student offering a *helping hand* to their professors in exchange for preferential treatment or better grades. Osipian (2008) as well as Chapman and Lindner (2016) point out that reciprocity in the sense of an exchange of favors is just as much a common form of HE corruption as are monetary forms of bribery. *White bribery* is the subtlest form of HE bribery because neither goods nor reciprocal non-monetary services are exchanged for being granted a favor. In contrast, the briber (the student, in this example) uses emotional stimuli as a means to strategically manipulate the other person who is in power (i.e., a professor or lecturer) to their advantage. In distress or if stakes are high, some people will go as far as to establish dishonest relationships to achieve their goal but softer forms using emotional influence tactics—such as crying, begging, and telling (fake) emotional family stories to cause compassion—can also be subsumed under *white bribery* in HE (Chapman & Lindner, 2016; Osipian, 2007). One of the great challenges of studying unethical behavior—of which bribery is a clear example—is that it is subject to social desirability bias (Randall & Fernandes, 1991) which might guide students behavior, so that it is logical to hypothesize that:

#### Hypothesis 1 (H1)

Students are less likely to accept the use of darker shades of bribery (i.e., grey and black) compared to lighter shades (i.e., white) bribery.

This study takes the briber as the central point of interest since we are, firstly, especially interested in how HE students behave in emotionally challenging, high-stake situations in which students may have the opportunity to offer a bribe to influence their lecturers’ decisions and, secondly, because the common situation of a student–lecturer consultation taking place without other witnesses (e.g., when students inquire to consult their lecturers about their exam grade) creates an especially vulnerable space of discretion that may be abused to mantle acts of bribery. den Nieuwenboer and Kaptein (2008) and Heyneman (2014) argue that bribery is especially prevalent in contexts in which supervisors’ power of sanctioning misconduct is diminished by a lack of transparency and in which members are stressed and hence feel pressured into committing unethical behavior.

The scientific discourse identifies diverse antecedents of bribery. From a macro perspective, HE bribery is rooted in the socio-cultural, economic, ethical, and institutional environment (Osipian, 2007; Ramdani & van Witteloostuijn, 2012). Although empirical evidence for Germany, Belgium, and the Netherlands is still scarce (with the exception of De Waele et al. (2021)), quantitative and qualitative research from Russia (Osipian, 2007; Petrov & Temple, 2004) and the countries of former Yugoslavia (Sabic-El-Rayess & Mansur, 2016) show that besides mundane monetary transactions (i.e., black bribery), reciprocal bribery in the form of informal quid-pro-quo (i.e., grey bribery) or favoritism (i.e., white bribery) is still a very prevalent practice in HE in these countries. Studying bribery in HE-contexts in Africa, Australia, China, India, and Russia, Mohamedbhai (2016)—in line with Downes (2017)—presents examples such as monetary transfers in exchange for a Ph.D. title, favoritism in the form of dubious appointments of professorships, and the extortion of money for handouts and grades.

Prior research by Martin et al. (2007) and Jávor and Jancsics (2016) emphasize the critical importance of individual micro-level attributes for the likelihood of individuals choosing to actually engage in bribery. Individual characteristics such as age, gender, and education, but also personal risk preferences and psychological and motivational factors—particularly *study-related stress* and *personal values*—have a decisive influence on the likelihood that an individual will offer and/or accept bribes (Alatas et al., 2009; Nichols & Robertson, 2017). Surprisingly, there is only scant research addressing these micro-level factors in the context of HE to-date. In the next section, we focus on two of the most important micro-level factors and their potential effect on students’ likelihood to bribe in detail: Study-Related Burnout and their Commitment to the Public Interest in detail.

### Study-Related Burnout

Burnout is a severe and increasingly prevalent condition among university students (Lin & Huang, 2014; Portoghese et al., 2018; Salmela-Aro & Read, 2017). With the Bologna reforms in Europe in the early 2000s, students are faced with growing demands, especially with a higher (perceived) workload and a higher frequency of testing, which may have devastating effects on students’ emotional, social, and physical wellbeing, implying that desperate measures such as bribing lecturers might become a more wide-spread phenomenon in the future (Lin & Huang, 2014; Portoghese et al., 2018; Salmela-Aro & Read, 2017). Moreover, recent studies from authors such as Fernández-Castillo (2021) and Jiang (2021) demonstrate that additional challenges related to the Covid-19 pandemic resulted in dramatically high levels of burnout among students, increasing anxiety and fewer social interactions, feelings of desperation and decreased capacity to cope with study workload and uncertainty.

Earlier studies by Koeske and Koeske (1991) and Jacobs and Dodd (2003) revealed that study-related burnout is associated with a substantially higher likelihood of adverse study outcomes such as higher intention to quit, poor academic performance (Salanova et al., 2010; Schaufeli et al., 2002), and low coping effectiveness, which might promote desperate measures and unethical behavior (Dwyer & Cummings, 2001; Gan et al., 2007). These problems are not restricted to the context of European HE but affect university students worldwide. For instance, in a cross-sectional and longitudinal cohort study of more than 4000 students in the U.S., Dyrbye et al. (2008) found that 49.6% of respondents suffered from symptoms of burnout, which were also associated with severe psychological strains such as suicidal ideation (11.2% of respondents).

Unsurprisingly, the effects of study-related burnout in HE have recently gained considerable scientific attention (Dyrbye et al., 2008; Jacobs & Dodd, 2003; Koeske & Koeske, 1991; Neumann et al., 1990; Salanova et al., 2010; Stoeber et al., 2011) but its key foundations date back to Freudenberger (1974). In his pioneering work, Freudenberger (1974) conducted case studies with volunteers engaged in health centers that treated people for drug and alcohol abuse to explore the specific demands of these volunteers’ engagement. Freudenberger (1974) defines the concept of burnout as an amalgamation of various negative symptoms such as exhaustion, deprivation, headaches, irritation, and frustration that were all related to the strains of his sample’s challenging voluntary work. Later, Maslach et al. (1986) and Maslach and Leiter (2008) developed the concept of burnout further by defining it as a syndrome of emotional exhaustion, depersonalization, and reduced personal ability to cope with job and life demands. In this context, it is important to note that burnout specifically affects people who do not suffer from clinical psychological disorders (Schaufeli & Enzmann, 1998). The current consensus is that burnout comprises three different but interacting dimensions: (1) *exhaustion*, i.e., a person’s fatigue, (2) *cynism*, i.e., a person’s indifference towards work, and (3) professional *efficacy*, which encompasses the loss of both social and non-social aspects of occupational accomplishments (Leiter & Schaufeli, 1996).

There are many reasons that explain why people develop burnout symptoms but the existing body of scholarship points out that workload does not solely drive this development (Leiter & Schaufeli, 1996). Instead, developing burnout is especially likely in contexts in which individuals experience substantial levels of emotional stress in executing their tasks, high personal engagement and identification with the task, and in which individuals’ perceived locus of control is relatively low (Schmitz et al., 2000)—a situation typically for students in HE. Burnout has gained considerable attention in the research field of human resource management, but in many cases findings are transferable to the context of HE (Jacobs & Dodd, 2003): Even though students are (mostly) not formally employed by their universities, following a structured study program encompasses coercive activities such as mandatory class attention and submitting scheduled assignments that can be very well considered as work (Stoeber et al., 2011). Yet, research on the adverse effects of burnout on (un-)ethical behavior in the research field of HE remains fairly limited. Based on a large sample of both students and lecturers in the U.S., Misra et al. (2000) found that study-related stress invoked strong negative emotional responses and symptoms that are significantly associated with burnout, varying from severe fear, anxiety, worry, or anger to crying, and to abusing themselves and others physically and emotionally. Ross et al. (1999), Jacobs and Dodd (2003), and Robotham and Julian (2006) provide quantitative evidence in which increased (perceived) workload in class and getting lower grades than anticipated are identified as major sources of stress, potentially leading to burnout and, consequently, deviant behavior as an (often desperate) coping mechanism (Jacobs & Dodd, 2003).

For instance, Ceschi et al. (2016) and Jacobs and Dodd (2003) found an empirical link between overwhelming job demands, burnout, and deviant behavior, such as bribery (Pulich & Tourigny, 2004). Penney and Spector (2005), Robotham and Julian (2006), and Kalliath et al. (2000) also provide empirical evidence linking deviant behavior with burnout because individuals suffering from burnout feel more depressed, more anxious, more emotionally erratic, and lack self-esteem (Bianchi, 2018; Connelly & Ones, 2008). Prior studies show that such negative affectivity and burnout are also strongly correlated with a higher likelihood for engaging in unethical behavior to cope with undesired events such as failure in important tasks (Penney & Spector, 2005; Robotham & Julian, 2006). Following these streams of prior research, we assume that student burnout might be directly related with higher chances of acting corruptly, especially if individuals are agitated about their current study experience, e.g., in situations of exam failure. Consequently, we hypothesize that:

#### Hypothesis 2 (H2)

Students are more likely to engage in bribery if they are affected by study-related burnout.

### Commitment to the Public Interest

Yet, students’ decision on whether or not to engage in unethical behavior—even if they suffer from burnout—might be influences by other, value-related motivations nested within the individual, especially their *commitment to the public interest*. A large body of scholarship grounded in the theory of planned behavior argues that personal values, ethics, and pro-social motives play an important role in guiding individual behavior especially when being faced with tough decisions (De Waele et al., 2021; Eisenberg, 2000; Moore et al., 2012; Ripoll, 2019). Developing a strong moral code directed toward the immediate and long-term interests of society as a whole assists individuals in self-regulating their actions toward honest and socially desirable behavior so that they become less likely to engage in bribery and other forms of corrupt behavior (Ajzen, 1991; Davis & Welton, 1991; Glover et al., 1997; Olsen et al., 2019). Consequently, we postulate that students suffering from study-related burnout are more likely to engage in bribery (Everall & Paulson, 2004) but that commitment to the public interest moderates this likelihood, assuming that students who feel emotionally drained by their studies would still refrain from engaging in bribery if they held a strong public values-related moral code.

One potential explanation on why individuals with high moral standards may be less likely to engage in desperate measures such as bribery is provided by Fritz et al. (1999) and Wright et al. (2016) who observed that individuals guided by high ethical standards were more committed to the public interest in general. This commitment enables them to resist exploiting opportunities for selfish reasons more effectively especially if serving their self-interest is disadvantageous to society. This indicates that an individual’s level of commitment toward the greater interests of society might play an important role in explaining why some students engage in bribery and others do not. Consequently, we assume that students with a high CPI are less likely to engage in bribery even if they experience symptoms of burnout. We hypothesize that.

#### Hypothesis 3 (H3)

The relationship between burnout and students’ likelihood to bribe is moderated by students’ commitment to the public interest.

## Materials and Methods

### Quasi-experimental Research Design

As a very delicate issue, bribery is hard to measure because respondents are likely to consciously or unconsciously conform to norms of social desirability, hence biasing their response to explicit questions related to their likelihood to bribe and to accept bribes even in the anonymity of online surveys (Petrov & Temple, 2004). Quantitative quasi-experiments^1^ using vignette-based treatments are a particularly valuable remedy for this problem because they help reveal the (latent) mechanisms that determine students’ likelihood to engage in bribery while circumventing this response bias in an elegant way: Vignettes are stimuli in the form of narrative scenarios that ask participants to imagine being *another* person, who has to act and make decisions within a certain context as specified within the narrative of the vignette (Hughes & Huby, 2004). By asking respondents to state what this other person would or should do, effects of social desirability bias are greatly reduced because the (implicit) psychological burden of being the singled-out decision maker is diminished for the respondent. Thus, vignettes have the power to systematically manipulate and trigger context-dependent behavior at high degrees of both internal and external validity (Aguines & Bradley, 2014).

The current study involves three quasi-experimental vignette treatments that differ regarding the information given to describe the shade of bribery (*white*, *grey*, and *black bribery*; see Online appendix A.1 for full detail). The vignettes were carefully designed by an international team of researchers to represent Heidenheimer’s (2009) and Ramdani and van Witteloostuijn’s (2014) three shades of bribery, ranging from *white* to *grey* and to *black* forms of bribery but within the specific context of HE. Each respondent randomly received two out three treatment vignettes in random order. Randomization offers the opportunity for causal inference, while inhibiting order effects (Meyer et al., 2017). Our treatment comprises scenarios in which respondents are in the active role of a student proposing a specific form of a bribe to a lecturer in exchange for the reconsideration of an important exam score for a failed exam. The first vignette represents *white bribery* in that the form of bribery is an emotional influence tactic aimed at inducing compassion and empathy (Batson et al., 1995). The student begs, cries, and gets emotional in order to persuade the lecturer to reconsider the grade. The second vignette involves *grey bribery* in that the student reciprocally offers a non-monetary service (a helping hand) in exchange for the lecturer reconsidering their grade. The third vignette represents the most commonly exposed form of bribery (*black bribery*) by involving a monetary transaction that is offering the classic brown envelop with €500 in exchange for a pass.

The external validity of this vignette design and treatment procedure was corroborated with an expert panel—as suggested by Gould (1996)—comprising both lecturers/professors and students of the faculties in which our samples were raised. Adequate pretests of the treatment stimuli were conducted before the experiment was rolled out (Wilson & While, 1998). Prior studies on corruption-related issues using similar (quasi-)experimental study designs and vignette-based framing stimuli found small to medium-sized effects, see, e.g., Weißmüller et al. (2020) or Ripoll and Ballart (2020). In the prospect of small to medium-sized effects (Cohen’s *d* ≤ 0.3; power = 0.8; α = 0.05), samples should comprise at least *n* = 176 respondents (Ellis, 2010), which has been achieved for each sample.

Respondents were randomly assigned to two out of three bribery vignettes to reduce the absolute number of participants needed while guaranteeing a satisfactory high amount of treatment variance. Treatment randomization is an essential requirement for research seeking to infer causal relations (Meyer et al., 2017). The vignettes were designed with due diligence following the suggestions by Hughes and Huby (2004) to make sure that the treatments are equally reliable, valid, logical, and comprehendible for the specific context of HE and for the specific target group of respondents (i.e., university students). The balance between treatment groups was strictly controlled for, with success (see Table 1). In total, the study design consists of four parts: A short introduction, a socio-demographic questionnaire with control variables (age, gender, religious beliefs, and field of study), independent variables, the vignette-treatment and dependent variable, and, lastly, a short debriefing.

**Table 1:** Descriptive sample statistics

Sample	Germany	Belgium	The Netherlands
N	211	220	193
Vignette treatment^a^			
Treatment 1: *white* bribery	33.8%	34.7%	34.2%
Treatment 2: *grey* bribery	33.8%	34.8%	31.4%
Treatment 3: *black* bribery	34.2%	34.9%	30.8%
Burnout	3.02 ± 0.87	3.01 ± 0.51	3.16 ± 0.56
Commitment to public interest (CPI)	5.63 ± 1.06	5.78 ± .94	5.50 ± 1.10
Gender, male (*n*)	45.2% (95)	48.2% (104)	48.2% (93)
Age in years	25.8 ± 4.8	22.5 ± 3.7	21.1 ± 2.8
Religion (*n*)			
Non-believer	40.8% (86)	49.6% (109)	67.7% (130)
Catholic	14.7% (31)	40.0% (88)	20.7% (40)
Protestant	33.7% (71)	2.3% (5)	6.7% (13)
Muslim	6.6% (14)	5.9% (13)	0.5% (1)
Jewish		0.5% (1)	0.5% (1)
Buddhist		0.5% (1)	1.6% (3)
Other	4.3% (9)	1.4% (1)	2.6% (5)
Field of study (*n*)			
Business administration	35.6% (75)	46.8% (103)	40.1% (79)
Socioeconomics & economic policy	9.9% (19)	10.0% (22)	31.3% (66)
Political science	3.6% (7)	7.3% (16)	5.7% (12)
Business engineering		24.1% (53)	4.3% (9)
Other social sciences	47.7% (92)	11.8% (26)	21.3% (45)

Items are either reported with geometric means and standard deviations (*M* ± *SD*) or proportions (%) and frequencies (*n*)

^a^Treatment distribution controlled for balance with two-tailed *t*-tests both within and between studies; all non-significant

### Sampling Procedure

Data were raised with a voluntary online survey among university students in summer 2017. The study was conducted in several waves at the faculties for business, economics, and social sciences of two large Dutch, one Belgian, and one German university. All institutions were selected because they represent particularly typical specimen of state-funded public universities offering the full canon of study fields (*full universities*) and large student samples potentially representative for the full student population in these countries. We selected these three Western European countries because the HE sector of Germany, Belgium, and the Netherlands are highly comparable: They apply the same type of admission system into HE to guarantee standardized admittance procedures for student selection, and they apply comparable standards regarding bureaucratic and academic rigor as regulated by the respective public code of law in each of the three study countries (Haj et al., 2018). Potential participants were invited through an e-mail distributed among their respective faculties. Respondents were incentivized with the possibility of winning one of five considerable gift vouchers (1 × €250, 1 × €150, and 3 × €50) for a popular online retailer in each country. The experiment was programmed and hosted with the software Qualtrics and it was distributed via e-mail invitation.^2^ The final sample comprises *N* = 624 respondents, 53.2% of which are female. Respondents are on average *M* = 23.2 (*SD* = 4.4) years old, predominantly nonreligious (52.1%), pursuing a variety of business and society-related degrees, predominantly business administration (41.1%) (see Table 1 for more detail). The resulting dataset was strictly stratified by excluding any observations with missing data and, consequently, comprises only complete responses.

### Dependent Variable: Acceptability of Bribing (BRIBE)

We use De Waele et al.’s (2021) four-item measure on the *acceptability of bribing (BRIBE)* as our main dependent variable. This measure asks respondents to indicate how likely they were to act as described in a corruption-related vignette (see Online appendix A.1 for more detail) using four dimensions: *likelihood*, *justification*, *affect*, and *mistake* (reversed), which are coded as five-point Likert-type items ranging from 1 = *absolutely disagree* to 5 = *absolutely agree*. The four dimensions are mean sum-scored to create *BRIBE*. We control the validity of this aggregation procedure by conducting an exploratory factor analysis (*varimax* rotated with Kaiser normalization for item correlation, *Chi*^*2*^ (6) = 2,622.98, *p* < 0.000; factor item uniqueness ranges from *U* = 0.27–0.46; Kaiser–Meyer–Olkin *KMO* = 0.83), which confirmed high internal construct validity. The derived factor model is well specified and shows that the four items strongly and significantly load onto one single underlying factor (Cronbach’s *α* = 0.874), indicating high external construct validity of the variable *BRIBE* with its four highly inter-correlated components. *BRIBE* is normally distributed across all treatment conditions [tested with Shapiro–Wilk; vignette 1: *W*(409) = 0.991, *p* = 0.015; vignette 2: *W*(417) = 0.954, *p* = 0.000; vignette 3: *W*(415) = 0.892, *p* = 0.000] and, thus, allows for linear regression analysis. As a control variable, respondents were asked to rate how realistic they found each scenario. Following recommendations by Krosnick and Presser (2010), we use an even four-point Likert-type single item, ranging from 1 = *very unrealistic* to 4 = *very realistic.*

### Burnout Scale

We use Schaufeli et al.’s (2002) well-established *burnout scale for university students* to assess the role of study-related stress as a factor influencing the likelihood that students accept the use of bribery. Schaufeli et al.’s (2002) scale is the result of a rigorous multi-national replication study based on the *Maslach Burnout Inventory* (Maslach et al., 1986) in a special adaption for students in HE. The scale measure is characterized by both high construct validity and high external reliability and consists of in total 15 seven-point Likert-type items clustered within three underlying dimensions (*exhaustion*, *cynicism*, and *professional efficacy*). In the current study, we use the scale as a global, compound measure that does not discriminate between the three sub-dimensions because all three of them are equally relevant for students’ study-related burnout and its relation to engaging in unethical behavior.

### Commitment to the Public Interest

We measure respondents’ commitment to the public interest (*CPI*) with Kim et al.’s (2013) well-established and internationally validated scale on public service motivation (PSM) in which CPI is one central dimension. Kim et al.’s (2013) full scale comprises four sub-dimensions to explain why some people are more motivated to engage in activities that are beneficial to the public interest (Grant, 2008; Perry & Wise, 1990). From these sub-dimensions—namely: *compassion*, *interest in policy-making*, *self-sacrifice*, and *commitment to the public interest*—we use *commitment to the public interest* (CPI) as a proxy to determine how individuals’ ethical standard might inhibit or escalate their likelihood to bribe. CPI is measured as the weighted geometric mean of three Likert-type statement items with response values ranging from 1 (= *absolutely disagree*) to 7 (= *absolutely agree*). Explicitly, these items asked respondents to indicate their personal opinion on (1) the relevance of civic duty, (2) the relevance of public service in general, and (3) the relevance of ethics in public institutions such as universities.

### Probability Discounting Questionnaire

Since most shades of bribery are illegal and violate the common ethical principles and values of HE, offering bribes is a risky and psychologically stressful endeavor. Consequently, it is important to control for individual differences regarding risk attitudes between study participants. We assess individuals’ risk propensity with Madden et al.’s (2009) *Probability Discounting Questionnaire*, a behavioral measure that estimates revealed risk propensity based on responses to a systematic and randomized set of 30 economic trade-off tasks. Payouts are hypothetical, but Madden et al.’s (2009) measure is very reliable in predicting not just preferences but also real choice behavior under risk (Green & Myerson, 2004), while at the same time being very robust against conscious manipulation. Using Weißmüller’s (2021) aggregation algorithm, the questionnaire results in one characteristic discounting parameter (*h*), which describes individual students’ likelihood to act risk-averse or risk-affine, respectively. The parameter *h* is exponential in scale and was, consequentially, centralized by taking its logarithm. Since higher discounting parameter values indicate that respondents devalue risky options more strongly, individuals with ln(*h*) > 0 are characterized as risk-averse.

### Control Variables

We complement our survey with socio-demographic control variables to (a) control for sample balance and (b) because some may be indicative for deviant and unethical behavior. We capture respondents’ gender, age, community of faith (Conroy & Emerson, 2004; Randolph-Seng & Nielsen, 2007), and field of study (Alatas et al., 2009; Nichols & Robertson, 2017).Prior research by Conroy and Emerson (2004) indicates that individuals who saliently associate with a community of faith are less likely to engage in deviant behaviour in general (Randolph-Seng & Nielsen, 2007) and that religious HE students are particularly less likely to engage in bribery and academic misconduct (Yu et al., 2017). In accordance with Holland’s (1992) theory of vocational preferences, a large number of studies—e.g., by Ekehammar et al. (1987), Hackett and Lent (1992), and Olsen et al. (2019)—show that students self-select into different fields of studies (e.g. economics vs. social sciences) not only based on their desired professional career opportunities in their later lives but also because students associate different schools of thought with different study fields (e.g. a more welfare-oriented behavioural paradigm with sociology as compared to a more self-serving rationale with economic and management studies). Students, hence, self-select in accordance with their ethical and socio-political attitudes to maximize person-environment fit (Ekehammar et al., 1987; Pike, 2006). Consequently, we use study field and religion as control variables to make sure that our samples are comparable cross-nationally and that variance is not nested unobserved in these latent factors.

### Model Estimation

Because study participants always responded to *two* vignettes, we conducted a linear regression analysis clustered at the subject level to ascertain that standard errors are robust against heteroscedasticity.^3^ Consequently, the number of pooled observations in the regression model amounts to 1,241 observations nested in *N* = 624 individuals. We estimate three regression models in total. The direct effects model (Model I) is specified as follows:

$$BRIBE= {\beta }_{1}Grey+ {\beta }_{2}Black+{\beta }_{3}Realism+ {\beta }_{4}Burnout+{\beta }_{5}CPI+ {\beta }_{6}Risk Aversion+{\beta }_{7}Age+{\beta }_{8}Female+{\beta }_{9}Country+\varepsilon$$

Model I tests the effect of study-related stress (*Burnout*) on the likelihood of bribing (*BRIBE*) while controlling for three different shades of the bribery treatment (*grey* and *black; white bribery* serving as the default category) to test H1 and H2. Based on prior empirical research pointing out that individuals’ personal characteristics influence their likelihood of engaging in acts of bribery (Glover et al., 1997), this base-line model includes a series of control variables to guarantee high ecological validity of the model (i.e., respondents’ individual revealed *risk propensity*, their *age*, their *gender* (with *female* set as the arbitrary default), and a binary indicator for high (i.e., larger than average) perceived *realism* of the treatment condition). The pairwise correlation matrix for all study and control variables is presented in Online appendix A.3.^4^ In a second and third model (Model II and Model III), we subsequently add interaction terms: firstly, between treatments and Burnout to explore H2 (Modell II) and, secondly between CPI and burnout to investigate H3 (Model III)*.* In the following section, we first analyze each country’s sample individually and then pool the data for a combined model in which Germany arbitrarily serves as the reference category to investigate cross-country effects.

## Results

### Study 1: Germany

The data of study 1 comprises responses by *n* = 211 participants (54.8% female) who are on average *M* = 25.84 (*SD* = 4.82) years old, mainly non-religious (40.8%) or of protestant faith (33.7%), and who predominantly study business administration (35.6%) or other social sciences (47.7%) at a large public university in Germany. Participants score average on Schaufeli et al.’s (2002) burnout scale (*M* = 3.02, *SD* = 0.87), hold relatively high levels of CPI (*M* = 5.63, *SD* = 1.06), and are revealed to be relatively risk averse (*M* = 0.62, *SD* = 0.59) but with a high degree of variance within the sample. For this sample, Schaufeli et al.’s (2002) burnout scale is highly reliable and robust with Cronbach’s α = 0.86 and resulted in a very satisfactory level of inter-item covariance (IIC) of 71.5% on average. Factor analysis on the three items of CPI confirms that all items are highly correlated and load unto one single underlying factor (Cronbach’s α = 0.72; average IIC = 0.818; Bartlett’s test for sphericity: Chi^2^ (3) = 296.25, *p* < 0.000; all mean KMO > 0.61), indicating high measurement reliability.

Robust linear regression analysis on *BRIBE* (clustered at the level of the individual for conditional contribution; see Table 2) shows that the contextual treatment (i.e., darkening shades of bribery; grey: *β*_*I*_ = − 0.441, *p* = 0.000; black: *β*_*I*_ = − 0.681, *p* = 0.000) and the perceived realism of the treatment vignettes (*β*_*I*_ = 0.456, *p* = 0.000) created a substantial amount of variance which adds to the robustness of our findings. Since respondents differentiate sharply between the three shades of bribery and are substantially more likely to engage in lighter shades (*β*_*I*_ = − 0.441, *p* = 0.000), H1 cannot be rejected. H2 postulates that students are more likely to engage in bribery if they are affected by burnout. Neither Model I nor Model II indicate that higher levels of burnout are associated with a higher likelihood of offering bribes (*β*_*I*_ = 0.066, *p* = 0.220), neither directly nor by moderation. Consequently, H2 has to be rejected. However, we note that the relationship between *BRIBE* and burnout is indeed positive, as hypothesized. In contrast, Model I reveals that higher commitment to the public interest is directly and negatively associated with students’ likelihood of engaging in acts of bribery (*β*_*I*_ = − 0.078, *p* = 0.062) but—contrary to H3—Model III shows that this effect is a direct (albeit weak) effect rather than being filtered through an interaction with burnout (*β*_*III*_ = 0.046, *p* = 0.370). Consequently, H3 has to be rejected for study 1.

**Table 2:** Regression analysis on BRIBE by country study

		Germany			Belgium			The Netherlands			Pooled data		
		I	II	III	I	II	III	I	II	III	I	II	III
*H1*	*Treatment effect*												
	White bribery	*Reference category*			*Reference category*			*Reference category*			*Reference category*		
	Grey bribery	− .441***	− .443***	− .675†	− .624***	− .625***	− 1.627*	− .481***	− .480***	− .939†	− .533***	− .533***	− .983***
		(.12)	(.12)	(.35)	(.11)	(.11)	(.56)	(.11)	(.11)	(.56)	(.06)	(.06)	(.25)
	Black bribery	− .681***	− .680***	− 1.137**	− .900***	− .901***	− 1.793**	− .700***	− .699***	− .948***	− .799***	− .798***	− 1.275***
		(.12)	(.12)	(.33)	(.10)	(.10)	(.53)	(.12)	(.12)	(.48)	(.06)	(.06)	(.23)
	Realism	.456***	.457***	.453***	.421***	.420***	.423***	.458***	.458***	.457***	.427***	.427***	.426***
		(.06)	(.06)	(.06)	(.05)	(.05)	(.05)	(.05)	(.05)	(.05)	(.03)	(.03)	(.03)
	*Two-way interaction*												
*H2*	Burnout × grey bribery		.078			.335^†^			.144			.147^†^	
			(.10)			(.18)			(.18)			(.08)	
*H2*	Burnout × black bribery		.151			.297^†^			.078			.155*	
			(.10)			(.17)			(.15)			(.07)	
*H3*	Burnout × CPI			.046			− .115			.032			.018
				(.05)			(.08)			(.06)			(.04)
	*Independent variables*												
*H2*	Burnout	.066	− .192	− .008	.061	.730	− .165	.117	− .063	.041	.084*	− .02	− .019
		(.05)	(.29)	(.08)	(.08)	(.48)	(.14)	(.08)	(.35)	(.12)	(.04)	(.22)	(.06)
*H3*	CPI	− .078^†^	− .225	− .078^†^	− .040	.301	− .039	− .083*	− .183	− .08*	− .070*	− .127	− .069**
		(.04)	(.16)	(.04)	(.04)	(.24)	(.04)	(.03)	(.19)	(.03)	(.02)	(.12)	(.02)
	Risk aversion	.089	.08	.087	.015	.016	.012	− .043	− .043	− .042	.017	.016	.018
		(.08)	(.08)	(.08)	(.06)	(.06)	(.06)	(.06)	(.06)	(.06)	(.03)	(.03)	(.08)
	Age	.025**	.024**	.025**	.011	.009	.011	− .004	− .004	− .003	.010^†^	.001^†^	.010^†^
		(.01)	(.01)	(.01)	(.01)	(.01)	(.01)	(.01)	(.01)	(.01)	(.01)	(.01)	(.01)
	Female	− .039	− .043	− .035	− .168*	− .164*	− .170**	− .124	− .125	− .126	− .142***	− .142***	− .140**
		(.10)	(.10)	(.10)	(.07)	(.07)	(.07)	(.08)	(.08)	(.08)	(.05)	(.05)	(.05)
	German										*Reference category*		
	Belgian										− .075	− .076	− .075
											(.07)	(.07)	(.07)
	Dutch										− .063	− .064	− .062
											(.06)	(.06)	(.06)
	Intercept	.921*	1.783^†^	1.157*	1.385**	− .568	2.061***	1.651***	2.204*	1.880***	1.486***	1.811**	1.806***
		(.44)	(.99)	(.50)	(.44)	(1.44)	(.58)	(.38)	(1.07)	(.44)	(.24)	(.69)	(.28)
	*Observations*	349	349	349	382	382	382	372	372	372	1169	1169	1169
	*F*	49.33***	46.70***	41.21***	60.78***	53.22***	49.64***	57.34***	51.16***	46.60***	125.86***	115.70***	107.33***
	*VIF *^a^	1.27			1.25			1.29			1.43		
	*R*^2^	.465	.466	.468	.529	.533	.536	.484	.485	.486	.478	.478	.480
	Adj. *R*^2^	.452	.452	.452	.519	.521	.523	.473	.472	.471	.473	.473	.475

Linear regression estimates clustered at subject level for conditional contribution

Model I: direct effects; Model II and III: interaction effects; heteroscedasticity-robust standard errors in parentheses

^†^*p* < 0.10, *p < 0.05, **p < 0.01, and ***p < 0.001

^a ^Mean variance inflation factor (*VIF*): all *VIF* ≤ 2.19

### Study 2: Belgium

Study 2 was conducted at a large Belgian university and comprises data of in total *n* = 220 respondents (51.8% female; on average *M* = 22.47 ± 3.65) years old) who mainly study for degrees in business administration (46.8%) and business engineering (24.1%). Study participants are predominantly non-religious (49.6%) or of roman-catholic confession (40.0%). They report relatively high CPI (*M* = 5.78, *SD* = 0.94) and an average level of study-related burnout (*M* = 3.01, *SD* = 0.51). Across all vignette treatments, respondents in study 2 score below scale average on *BRIBE* (*M* = 2.03, *SD* = 0.97). Two-tailed *t*-testing reveals that the bribery vignettes create significant variance across the three treatment groups, with the likelihood to *BRIBE* strictly and transitively decreasing from the *white* (*M* = 2.65, *SD* = 0.94) to the *grey* (*M* = 1.86, *SD* = 0.85) and to the *black* bribery scenario (*M* = 1.56, *SD* = 0.78). This indicates a strong and robust treatment effect [*F*(1, 387) = 105.24, *p* = 0.000, *adj. R*^*2*^ = 0.213; *t* = − 10.26, *p* = 0.000, η^2^ = 0.215] and shows that H1 cannot be rejected. For this sample, the burnout scale is highly reliable and robust with Cronbach’s α = 0.85 and an acceptable level of IIC (47.1% on average). Factor analysis on the three items of CPI confirms that all items are highly correlated and load unto one single underlying factor (Cronbach’s α = 0.67; average IIC: 0.60; Bartlett’s test for sphericity: Chi^2^ (3) = 185.32, *p* < 0.000; all mean KMO > 0.62), indicating an acceptable level of measurement reliability.

Robust linear regression analysis on *BRIBE* (clustered at the level of the individual; see Table 2) reveals partially dissimilar results compared with study 1: The contextual bribery treatments (grey: *β*_*I*_ = − 0.624, *p* = 0.000; black: *β*_*I*_ = − 0.900, *p* = 0.000) and the perceived realism of the treatment vignettes (*β*_*I*_ = 0.421, *p* = 0.000) explain a substantial amount of variance and higher levels of burnout are, again, not significantly associated with a higher likelihood of offering bribes (*β*_*I*_ = 0.061, *p* = 0.424). In contrast to study 1, model II shows a strong bur only marginally significant positive interaction effect between students’ level of burnout and the shades of bribery (burnout × grey: *β*_*II*_ = 0.335, *p* = 0.066; burnout × black: *β*_*II*_ = 0.297, *p* = 0.089). This means that the Belgian students in our sample tend to be more likely to engage in bribery if they suffer from burnout but this effect is highly conditional on the type of bribe. With this caveat, H2 cannot be rejected. Regarding the hypothesized direct effect of CPI as an inhibitor of burnout-induced bribery (H3), we find no statistically significant direct (*β*_*I*_ = − 0.040, *p* = 0.268) or interaction effect (*β*_*III*_ = − 0.115, *p* = 0.148). Consequently, H3 has to be rejected for study 2.

### Study 3: The Netherlands

The results of study 3 are based on a sample of university students (*n* = 193; 51.8% female) mainly pursuing degrees in business administration (40.1%) and socioeconomics and economic policy (31.3%) at two large Dutch universities. Respondents are on average a little bit younger than respondents in studies 1 and 2 (*M* = 21.13, *SD* = 2.82), and predominantly non-religious (67.7%). They report above-average levels of study-related burnout (*M* = 3.16, *SD* = 0.56) and a relatively high level of CPI (*M* = 5.50, *SD* = 1.10). Similarly to study 1, the scale measures are highly reliable and robust (Burnout: Cronbach’s α = 0.88, average IIC = 58.5%; CPI: Cronbach’s α = 0.86, average IIC = 70.6%, Chi^2^ (3) = 258.69, *p* < 0.000, all mean KMO > 0.61).

The clustered robust linear regression models on *BRIBE* (see Table 2) reveal very similar results compared with both studies 1 and 2: The contextual bribery treatments created a substantial amount of variance (grey: *β*_*1*_ = − 0.481, *p* = 0.000; black: *β*_*1*_ = − 0.700, *p* = 0.000) and together with the perceived realism of the treatment vignettes (*β*_*1*_ = 0.458, *p* = 0.000) explain a high amount of variance. Respondents’ likelihood to *BRIBE* decreases transitively from white to black shades of bribery so that H1 cannot be rejected. We find a tentative positive relation between higher levels of burnout and a higher likelihood of offering bribes but this result is not statistically reliable (*β*_*I*_ = 0.117, *p* = 0.129) and neither are the interaction effects between treatment and burnout reported in Model II. In line with study 1 but contrary to the results of study 3, higher CPI is directly related with a lower likelihood of offering bribes (*β*_*I*_ = − 0.083, *p* = 0.016) but there is no interaction between CPI and Burnout (*β*_*III*_ = − 0.032, *p* = 0.606). H3 finds no support with data of study 3, rather CPI exerts a direct negative effect on the likelihood to bribe.

### Pooled Data

Pooling the data of all three country samples (*n* = 1169), linear regression analyses clustered on the level of the individual further substantiate the results presented in the previous sections, with respondents being linearly and transitively more willing to engage in lighter shades of bribery compared to darker shades (grey: *β*_*I*_ = − 0.553, *p* = 0.000; black: *β*_*I*_ = − 0.799, *p* = 0.000) so that H1 cannot be rejected. Higher levels of burnout are directly related to a higher likelihood of students being willing to engage in activities of bribery (*β*_*I*_ = 0.084, *p* = 0.025), supporting H2, and higher CPI is directly associated with a lower likelihood of *BRIBE* (*β*_*I*_ = − 0.070, *p* = 0.001). Both effects are rather small—with parts of this burnout-related effect channeled through an interaction with the type of bribe (grey × burnout: *β*_*II*_ = 0.147, *p* = 0.055; black × burnout: *β*_*II*_ = 0.155, *p* = 0.036). Consequently, neither H2 nor H3 can be rejected.

**Fig. 1:**
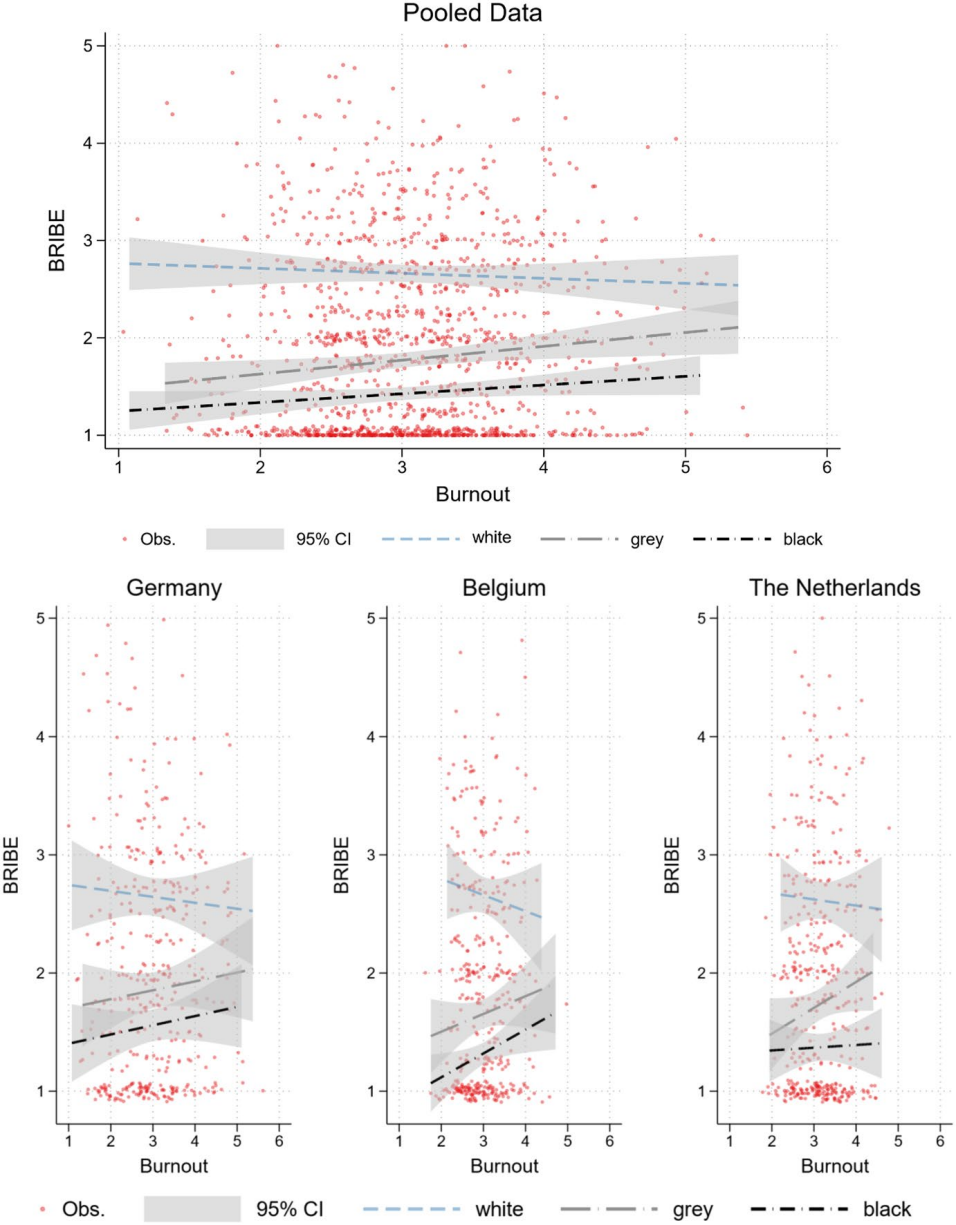
Fixed effects plot of Burnout on BRIBE, by treatment

The models indicate no substantial country effects underlining the high ecological reliability of our three country findings and the merit of the replication study design (Freese, 2007; Makel & Plucker, 2014). Across all three studies, we find that students are far less likely to accept the use of darker shades of bribery compared with lighter shades. Figure 1 illustrates the treatment effects in relation to respondents’ level of burnout by country in more detail. Comparing the three studies, we find that although respondents are much more accepting of the use of white bribery—i.e., psychological and emotional influence tactics—in contrast to grey and black bribery in general, this acceptability of white bribery decreases with growing levels of study-related burnout. In contrast, higher levels of burnout are associated with an *increase* in the acceptability of both grey and black bribery across all three country samples (see Fig. 1). Conversely, this also means that students who are less strongly affected by study-related burnout will be substantially more likely to use emotional influence tactics for their personal benefit compared with those students who experience more severe symptoms.

Curiously, and across all three studies, respondents who perceived the scenario presented in the vignettes as more realistic were actually *more* likely to accept the use of bribery as a means to improve their failed exams (*β*_*I*_ = 0.427, *p* = 0.000). This is an intriguing finding because it substantiates the high ecological validity of both the quasi-experimental procedure of this study and its findings, indicating that in these cases respondents were especially less likely to answer in a socially desirable way. Furthermore, in each country study, all regression models are well specified [*F*(df, 349–1169) = 41.21 − 125.86, *p* = 0.000] and explain a large share of variance (*adj. R*^*2*^ = 0.452 − 0.523), indicating robust and reliable findings. Multi-collinearity was not an issue (all *VIF* = 1.25 − 1.43). In summary (see Table 3), the empirical results show that the quasi-experimental replication approach used was successful in revealing actual intention to *BRIBE* but that one of the three hypotheses (H3) had to be rejected: Some shades of bribery are more likely to occur (especially emotional influence tactics), the likelihood of engaging in bribery increases with higher levels of burnout but this effect is in fact contingent on the type of bribe offered, and students’ commitment to the public interest is not a reliable factor that inhibits academic misconduct.

**Table 3:** Overview of findings

Hypothesis			Study 1	Study 2	Study 3	Pooled data	Interpretation
H1	(−)	Darker shade → BRIBE					Consistently negative and transitive from lighter to darker shades of bribery → H1 not rejected
		Grey vs. white treatment	− .441*** (.000)	− .624*** (.000)	− .481*** (.000)	− .533*** (.000)	
		Black vs. white treatment	− .681*** (.000)	− .900*** (.000)	− .700*** (.000)	− .799*** (.000)	
H2	(+)	Burnout → BRIBE	.066 (.220)	.061 (.424)	.117 (.129)	.084* (.025)	Consistently positive, but context (treatment) dependent → H2 not rejected
		Burnout × grey treatment → BRIBE	.078 (.457)	.335^†^ (.066)	.144 (.410)	.147^†^ (.055)	
		Burnout × black treatment → BRIBE	.151 (.140)	.297^†^ (.089)	.078 (.603)	.155^†^ (.036)	
Additional analysis	(−)	CPI → BRIBE	− .078^†^ (.062)	− .040 (.268)	− .083* (.016)	− .070** (.001)	Consistently negative
H3	(−)	Burnout × CPI → BRIBE	.05 (.144)	− .115 (.148)	− .032 (.606)	− .018 (.626)	Mostly negative effect but not statistically reliable; CPI and burnout exert effects in opposite directions; no significant interaction → H3 rejected

Analysis based on beta coefficients, with *p*-values in parentheses

^†^*p* < 0.10, *p < 0.05, **p < 0.01, ***p < 0.001

## Discussion

The findings confirm that students experiencing symptoms of burnout are more likely to use bribery as a means to influence their lecturers, for instance to pass failed exams. Pooled analyses indicate a robust and significant effect between burnout and the acceptability to bribe. This finding aligns with prior research by (among others) Penney and Spector (2005), Robotham and Julian (2006), and Reynolds et al. (2013) who found similar conditions among students to be positively correlated with a higher likelihood of engaging in unethical and deviant behaviors—of which bribery us a clear example—particularly when experiencing psychological burdens (Jacobs & Dodd, 2003; Portoghese et al., 2018; Salmela-Aro & Read, 2017; Stoeber et al., 2011). Across all three countries, the findings of the replication studies were mostly consistent, underlining the credibility and external validity of the findings brought forward (Freese, 2007; Hedges, 2019). However, in Belgium, the burnout effect was revealed to being relatively stronger compared with the studies conducted in Germany and the Netherlands. One explanation could be that, according to Hofstede (2003), the level of uncertainty-avoidance in Belgium is generally considered as comparatively high. Failing an important exam (i.e., the scenario in our vignette treatment) may signal a high amount of uncertainty. In this, the treatment may have resonated with relatively higher stimulus saliency for our sample of university students in Belgium, resulting in relatively stronger treatment effects and, hence, a higher willingness to engage in bribery compared to respondents from Germany and the Netherlands who generally score significantly lower on Hofstede’s (2003) measure of uncertainty avoidance.^5^

However, the relationship between bribery and burnout is strongly contingent on the type of bribery. Study participants in three countries revealed that they perceived engaging in white bribery and—to a certain extent—even grey bribery as a relatively well acceptable tactic to convince their lecturers to reconsider a failed exam. This finding does not only reveal that university students, indeed, distinguish sharply between different shades of bribery but also that lighter shades—especially emotional influence tactics (*white bribery*)—are hardly perceived as unethical behavior at all and are, hence, socially acceptable for most students in our samples. This is a troubling finding because it indicates that students are largely unaware that this kind of behavior is already a form of HE bribery: Even if (implicitly) regarded as socially acceptable, applying manipulative emotional influence tactics still aims at receiving illegitimate privilege compared to their fellow students who refrain from doing so, hence, undermining the principles of equal treatment and trust in the fairness of examination in HE.

Furthermore, findings show that students with high commitment to the public interest are only marginally less likely to engage in bribery. This is surprising and stands in contrast with classic predictions on the relevance of appealing to students’ ethical believes to direct individuals toward making socially acceptable decisions. Contradicting prior empirical research by, for instance, Trevino (1986), Ajzen (2001), Glover et al. (1997), and Ritter (2006), our findings are in line with arguments by Heyneman et al. (2008) and Heyneman (2014). In his essay on the corruption of ethics in HE, Heyneman (2014) points out that even though university students worldwide feel uncomfortable about engaging in study-related misbehavior—for instance by cheating in their exams and by bribing lecturers—those individuals who *do* engage in this academic misconduct will *still* report that they are satisfied with their behavior from an ethical perspective. This phenomenon resonates loudly with the theory of cognitive dissonance, a theory less frequently used in the context of HE but one that has been used for decades to explain deviant behavior in the context of organizations and work (Festinger, 1962; Moore, 2008). The cognitive dissonance theory suggests that individuals strive for internal psychological consistency in order to mentally function in the real world (Festinger, 1962). People who are aware of internal *in*consistencies are likely to experience psychological discomfort that will motivate them to reduce the cognitive dissonance by consciously or unconsciously rationalizing their behavior and, thus, justify it for themselves and others by either adding new parts to the cognition to fix the inconsistencies or by avoiding social situations that would result in discomfort and emotional and cognitive burden through exposure of the misbehavior (Festinger, 1962).

A second explanation of the relatively small correlation between holding high ethical standards and the likelihood of engaging in study-related bribery observed in the current study relates to the phenomenon of moral disengagement. Moral disengagement describes the conscious or unconscious process of dissociating individuals’ own behavior from the standards of morality they would normally deem legitimate, thus suspending the moderating influence of holding high ethical standards on behavioral self-regulation (Moore, 2008; Tsang, 2002).

The result that CPI is only marginally related with the likelihood of engaging in bribery emphasizes the weakness of merely reinforcing ethical appeals to prevent bribery and it illustrates the limitation of such appeals. This is a particularly important result for practice because it indicates that cases of bribery in student–lecturer consultation can hardly be prevented by moral appeals alone but that they should rather be addressed by making adaptations in procedural and organizational structures, resonating with recommendations by Denisova-Schmidt (2018). In practice, this can be achieved in a number of ways but we particularly recommend implementing a threefold strategy: First, developing and actively promoting explicit and transparent codes of conduct to enhance both students’ and lecturers’ awareness of the danger and different shades of HE. Second, the introduction of explicit criteria and procedures for handling cases of bribery to nurture a sustainable culture of transparency and to inhibit bribery intent by engagement (Fritz et al., 1999). Third, actively involving students, tutors, and lecturers in the development and implementation of anti-bribery policies also calls for mutual awareness of each other’s actions to reduce incentive and opportunities for misconduct (Zamaletdinov et al., 2016). For instance, practitioners seeking to reduce the likelihood of bribery when meeting with students wishing to discuss their exam results might want to ask another colleague to join them (four-eye principle) in critical situations to serve as an additional deterrent for students who are willing to offer bribes.

## Conclusion

The research design employed in this article directly responds to recent appeals by Petrov and Temple (2004), Osipian (2008), Makel and Plucker (2014), and Chapman and Lindner (2016) for replicating studies by using experimental study designs and it comes with a number of key methodological advantages. First, this design presents a novel approach in the research field of HE by using a quasi-experimental method on the issue of HE bribery, allowing the identification of treatment-related causal mechanisms (Meyer et al., 2017). Second, by replicating our study in three Western European countries, this study focused on countries in which bribery in HE is often (falsely) perceived as a marginal problem and, consequently, severely understudied (Chapman & Lindner, 2016; von Arnim, 2003), even though it is likely that these countries’ HE systems suffer from similar degrees of bribery as other OECD countries (Chapman & Lindner, 2016). Third, by replicating the study with three independent but comparable samples of university students in three countries, our empirical research strategy ensures high internal and external reliability and high validity of the findings by warranting precision and accuracy (Freese, 2007; Hedges, 2019) (Makel & Plucker, 2014). In terms of theory, it is the first article to integrate and test the hypothesized relationship between two important challenges in HE—bribery and study-related burnout—and to analyze this relationship from a student-centered, micro-level perspective (Glendinning et al., 2019).

The motivation for this study was to explore the connection between study-related burnout and bribery in a higher education context. Findings show that university students’ level of burnout is partially associated with their intent to bribe their lecturer for passing important exams, and that the use of emotional influence tactics is perceived as much more acceptable compared with other forms of bribery as a means to manipulate lecturers.

Like any empirical study, our study is subject to limitations. We use data from a vignette-based survey (quasi-)experiment and do not directly examine real-life behavior but behavioral intent. Yet, stated intentions to bribe still largely correlate with actual behavior in anonymous setups and, hence, grant very valuable insights into the delicate topic of HE bribery (Ajzen, 2001). Given the issue of social desirability, the effect sizes of the results might actually be under-reported, thus, calling for future research (Randall & Fernandes, 1991). Future studies might also want to manipulate other contextual aspects such as the effect of the four-eye principle (e.g., having a potential witness of the bribery act) or investigate the degree to which other motives and character traits influence students’ likelihood to bribe by using the BIG-5 personality inventory, for instance.

While behavioral intent is a good indicator for actual real-life behavior, more quantitative behavioral and qualitative observational research is needed to further substantiate the ecological validity of our results. Also, we did not explicitly control for grade point average (GPA) because data protection rules prohibited us from asking the students in our samples about this information since it could be used to indirectly identify individuals. Yet, prior research by McCarthy et al. (1990) and Stallman (2010) revealed that lower GPA is significantly related with psychological distress, mental health issues, and student burnout in particular. Consequently, we do encourage scholars conducting future replications of our study design with other student populations to include this indicator as a valuable control variable.

Furthermore, the current study solely follows the perspective of the agent offering a bribe. Consequently, this study cannot make assumptions about the extent to which the actions of one agent (the bribe-offerer) would effectively lead to a transaction between two agents (bribe-offerer and bribe-taker) since the viewpoint of the potential acceptor of this very bribe was not explicitly examined. Future studies conducting dynamic lab-based choice experiments will close this gap.

In the future, exact replications of this study’s design could be conducted in countries with a dissimilar socio-cultural perception of bribery to determine whether the effects revealed by the current study are idiosyncratic or generalizable. This is important because—although our main findings were replicated at large in three independent countries—our study essentially relies on convenience samples nested within specific universities because study participation was voluntary. While virtually all samples in social science research are convenience samples, Landers and Behrend (2015) point out that this sampling strategy has consequences for the generalizability of the findings derived from such data because convenience sampling with HE students imposes range restrictions that may lead to attenuation of effect sizes observed. For instance, the relationship between burnout and *BRIBE* observed in our data might actually be artificially deflated in comparison to if we had had the opportunity to conduct this study with the full student population at the partaking universities because individuals who show severe symptoms of study-related burnout might be comparatively more unable or unwilling to respond to the survey invitation compared with the general student population and they might, hence, be *under*represented. Yet, replicating our study in three countries is a partial remedy to this issue because it allows us to generalize *across* three convenience samples and show that there is, indeed, a systematic relationship between bribery and burnout in students. We assume that replications with full faculty-wide population-based samples will result in larger effect sizes (Freese, 2007; Landers & Behrend, 2015; Meyer et al., 2017).

The findings presented and discussed in this study are especially relevant for practice. We advise practitioners to not only focus on the more obvious shade of black bribery but to create awareness among their students and faculty for the more subtle forms of bribery such as emotional pleading or offering a helping hand because our study shows that individuals are much more tolerant towards and, hence, susceptible to those *white* and *grey* shades of bribery than to the *classic* brown envelop. Consequently, HE institutions could benefit from promoting awareness campaigns and practitioners’ workshops that enable lecturers to better recognize these influence tactics. Also, universities will benefit from educating students that such emotional influence tactics are inappropriate and potentially punishable in accordance to the institution’s ethical code of conduct. By engaging all stakeholders equally and by embracing ethical introspection, HE institutions can master the “shift from a mode of self-protection and denial to a mode of transparency and active engagement” (Heyneman, 2014, p. 5) masla.

## Supplementary Information

Below is the link to the electronic supplementary material.

Online appendix (DOCX 28 KB)

## Footnotes

1 We label the research design of this study as a *quasi*-experimental design because we only randomized the treatment across respondents. In a fully experimental setup, the different outcome-levels of the independent variable would also have had to be randomized as to strictly control for a-priori variance of this independent variable within treatment groups and to, hence, assign treatments in a balanced way across treatment groups. In the current study, this is tricky because the independent variable CPI is nested within individuals’ character. One possible solution would have been to conduct a pre-study measuring individuals’ levels of CPI and then—after a substantial temporal delay—invite students to the main wave of the experiment (multi-wave panel design). Unfortunately, within the scope of our research project, this was not possible because the ethical standards of using the samples at hand did not allow us to contact students directly for follow-ups in order to secure respondents’ full anonymity. Nevertheless, we encourage researchers planning to conduct replications of the current study to employ a multi-wave panel design if possible.

2 Specifically, in each study country, we used the e-mail distribution system of the three schools of business, economics, and social sciences that collaborated with us in the three countries, respectively. In Belgium, the e-mail was sent to 1000 bachelor students enrolled at a large public university in the Dutch-speaking part of the country. *N* = 289 students responded (response rate: 28.9%), of which *n* = 220 (relative completion rate: 73.0%) finished the survey (absolute completion rate: 22.0%). In Germany, the e-mail was sent to 2150 bachelor and master students at a large public university. These students had previously registered in the school’s e-mail list for voluntary participation in economic experiments. The students registered in this list amount to 35.3% of the total number of students enrolled at this school. *N* = 268 students responded (response rate: 12.5%), of which *n* = 211 (relative completion rate: 78.7%) completed the survey (absolute completion rate: 9.8%). In the Netherlands, data were raised by e-mailing 1,400 students in total at two large public universities. *N* = 232 students responded to this invitation (response rate: 16.6%), *n* = 193 (relative completion rate: 83.2%) of which complete the survey (absolute completion rate: 13.8%).

3 Online Appendix A.2 provides the results of extensive post-hoc analyses to control for order and spill-over effects potentially resulting from randomization-based latent secondary treatment-clusters between respondents. These results show that both the experimental setup and the randomization procedure are robust against these latent secondary treatment-clusters and that procedure-based order and spill-over effects are not an issue.

4 Pairwise correlation analysis reveals that respondents’ *study field* and religiousness (i.e., the variable *community of faith*) do not substantially correlate with *BRIBE*, the dependent variable of our study (study field: *ρ* = 0.02, *p* > 0.10; community of faith: *ρ* = 0.02, *p* > 0.10). Estimating the robust regression models including these two variables would decrease model fit [pooled: *F*(11, 583) = 113.74, *p* < 0.000]. Consequently, study field and community of faith were only used in the descriptive analysis of the samples to guarantee successful sample balancing and are not included in the main steps of analysis.

5 In Hofstede’s (2003, p. 151) large scale country culture study, respondents from Germany scored on average *M* = 53 on uncertainty avoidance, respondents from the Netherlands *M* = 45, and respondents from Belgium *M* = 80, which is one of the highest values of all countries studied, (values controlled for respondents’ age; *M* = 53, *SD* = 24; *min*. = 31; *max*. = 112).
