# Supplementary material for: Would you Bribe your Lecturer? A Quasi-experimental Study on Burnout and Bribery in Higher Education
Source: Res High Educ. 2021 Dec 2;63(5):768–96. doi: 10.1007/s11162-021-09669-1 (PMC8638650; doi:10.1007/s11162-021-09669-1)
Supplement: Supplementary file 1 — Online appendix (DOCX 28 KB) [file 11162_2021_9669_MOESM1_ESM.docx]

# Appendix (Supplementary Online Material)

A.1 Vignette Stimuli (English translation)

| 1. **Introduction to bribery scenarios [all study participants]:**   ‘Please imagine that you are a first-year student again who has just received his results for the end of term exams. You passed all courses but one. You failed to pass one very difficult course you really do not want to redo. The consequence is that your prerequisites in the next academic year become compromised and you are unable to participate in other courses so that chances are real that you will not succeed to obtain your degree within the foreseen four years.  Meanwhile, you informed the assistant of this course in order to receive written feedback. This feedback indicates that you achieved 9.4/20. You know that if you would have scored 9.5/20, your result would be rounded off to 10/20 so that you would have passed the exam and the study program of the first year would have been accomplished.  What would you do in the following two situations?’ | |
| --- | --- |
| 1. **Vignettes:**   Study participants randomly received **two** **out of three** vignette treatments, each followed by the five Likert-type scale factor items of the dependent variable *BRIBE*. | |
|  | 1. ***Emotional plea*: white corruption**   ‘You make an appointment with the lecturer of this course and inform him about your situation. Rumour goes that, in the past, the lecturer let himself be influenced in a personal conversation. After you became emotional, you ask the lecturer if he, due to the circumstances, would consider being a little bit milder with regard to your result so that you can finally succeed in this course. Your future relies on this.’ |
|  | 1. ***Car mechanic*: grey corruption**   ‘You make an appointment with the lecturer of this course and inform him about your situation. Rumour goes that, in the past, the lecturer let himself be influenced in a personal conversation. The lecturer is a little bit too late and apologizes. He experienced car trouble, which is very unfortunate for the reason that he has to leave for an important conference tomorrow. However, your father is a car mechanic. You offer your lecturer to repair the car, free of charge and with the highest priority, on the condition that your result is reconsidered.’ |
|  | 1. ***Brown envelop*: black corruption**   ‘You make an appointment with the lecturer of this course and inform him about your situation. Rumour goes that, in the past, the lecturer let himself be influenced in a personal conversation. You ask him to reconsider your score and therefore offer him an envelope with €500 in exchange.’ |

*Note.* Extensive codebooks in Dutch and German upon request.

**A.2 Additional Analysis on Order and Spill-over Effects**

In each study country, respondents were treated with two vignettes, which were drawn randomly out of three vignettes. Compared to a between-subject design in which each respondent would receive only one single vignette, this randomization approach dramatically reduces the number of respondents needed to achieve reasonable sample sizes to investigate treatment effects with respect to the anticipated effect sizes. Yet, this method of distributing the treatments could potentially confound the observed treatment effect on the main dependent variable *BRIBE* because showing two randomly drawn vignettes to each respondent results in latent second-order clusters between respondents based on the unique vignette order each of them received. For instance, the effect of receiving the white bribery vignette first followed by a grey bribery vignette second could relatively outweigh the effect of receiving two extreme conditions – for instance, in the form of first receiving the white bribery vignette followed by the black vignette.

The technical implementation of our quasi-experimental design allows us to identify three unique combinations of vignettes, as described in table A.2.1: *white* *&* *grey* (cluster *C1*), *black* *&* *white* (cluster *C2*), and *grey* *&* *white* (cluster *C3*). Hence, cluster *C2* represents the combination of receiving the two most extreme treatment conditions.

**Table A.2.1**: Descriptive statistics of *BRIBE* by latent second-order treatment clusters

| ***BRIBE*** | | | ***Obs.*** | ***Mean*** | ***SD*** | ***Min.*** | ***Max.*** |
| --- | --- | --- | --- | --- | --- | --- | --- |
| Cluster description | | |  |  |  |  |  |
|  | *C1* | White & grey bribery treatment | 412 | 2.106 | .928 | 1.000 | 4.750 |
|  | *C2* | Black & white bribery treatment | 407 | 2.044 | 1.038 | 1.000 | 5.000 |
|  | *C3* | Grey & black bribery treatment | 422 | 1.720 | .833 | 1.000 | 5.000 |

*Notes*: Pooled data; *BRIBE* values range: 1 = ‘very low’ to 5 = ‘very high’.

Mean comparison analysis (see table A.2.1) reveals only very mild cluster-based order effects within treatments, indicating that receiving a combination of the white and grey bribery treatment (*C1*) correlates with a higher likelihood of *BRIBE* compared with receiving a treatment cluster including the black bribery vignette (M*_C1_* > M*_C2_* > M*_C3_*), which is in line with both the hypothesized direction of effects in the study and with the results presented in the main analysis. Similar to the effects reported in the main analysis section, the effect of receiving a latent cluster of two extreme treatment conditions – the white and the black vignette (*C2*) – is associated with a substantial decrease in *BRIBE* but the effect is even larger if the black bribery vignette is combined with the grey bribery vignette (*C3*). This effect can be explained by the well-researched psychological effect of the negativity bias: A large body of research shows that negative stimuli are generally more salient than positive stimuli and, consequently, clusters that incorporate the socially less acceptable – i.e., black – form of bribery (*C2* and *C3*) are likely to result in lower likelihoods of *BRIBE*, indicating that the randomization approach resulted in a well-balanced treatment distribution. Since these findings are in line with our expectations, consequently, we find that vignette cluster-based spillover effects do not substantially confound the results of the current study, although mild cluster effects exist.

Since confidence intervals are relatively wide, we investigate the robustness of these mild cluster effects by conducting a series of two-tailed *t*-tests between the three clusters on the pooled data (see Table A.2.2).

**Table A.2.2**: Between-cluster differences of *BRIBE*

| ***BRIBE*** | |  | ***t*** | ***p*** | ***\|d\|*** |
| --- | --- | --- | --- | --- | --- |
| Cluster comparison | | |  |  |  |
|  | *C1 vs. C2* | [white & grey] vs. [black & white] | .909 | .364 | .064 |
|  | *C2 vs. C3* | [black & white] vs. [grey & black] | 4.943 | .000 | .345 |
|  | *C3 vs. C1* | [grey & black] vs. [white & grey] | 6.324 | .000 | .439 |
| Extreme cluster comparison | | |  |  |  |
|  | *C1 & C3 vs. C2* | [white & grey] or [grey & black] vs. [black & white] | -2.209 | .028 | .140 |

*Notes*: Clustered treatment effect; tested with two-tailed *t*-tests; effect sizes estimated with Cohen’s *d*-score (Welch-adjusted).

We find statistically significant but small differences between respondents who received the most extreme black and white bribery treatment (*C2*) and those who received the more moderate combination of the grey and black bribery treatment (*C3*) [*C2* vs. *C3*: *t* = 4.943, *p* = 0.000; |*d*| = 0.345] or those who received the white and grey bribery treatment (*C1*) [*C3* vs. *C1*: *t* = 6.324, *p* = 0.000; |*d*| = 0.439]; [*C1* & *C3* vs. *C2*: *t* = -2.209, *p* = 0.028; |*d*| = 0.140]. This makes a lot of sense since cognitive psychology research shows that being framed with a rather negative – i.e., black bribery scenario – or a rather positive – i.e., white bribery – treatment condition creates an implicit benchmark for the evaluation of the situation for respondents in consecutive choice scenarios. Although we would also expect a significant difference between being treated with the white and grey bribery treatments (*C1*) compared to being treated with the more extreme black and white treatment cluster (*C2*), two-tailed *t*-testing reveals no substantial differences in *BRIBE* (*t* = 0.909, *p* = 0.364; |*d*| = 0.064). This can be explained by the phenomenon that – compared with the white bribery scenario – both the grey and the black bribery scenario present scenarios that are less socially acceptable and which might, hence, trigger almost equally negative psychological benchmarks for evaluation.

Since the compound treatment effects of the latent between-subject vignette-clusters strongly resemble the findings in the main analysis, we conclude that the current experimental setup is robust against noise involuntarily induced by the randomization procedure-based order effects, and we, hence, conclude that order or spillover effects between vignettes were not a substantial issue.

In summary, we have great confidence in our findings but we do encourage scholars conducting future replications of the current study to recognize the methodological risk of involuntarily creating additional noise by using automatized randomization procedures that might result in latent vignette-clusters in the treatment distribution among respondents in our study. Although we do not find any substantial bias induced by these latent treatment clusters, future replication studies could, alternatively, use a pure between-subject design in which respondents receive, first, a non-affective neutral vignette to set a neutral benchmark across all respondents followed by, second, a single (*white*, *grey*, or *black*) treatment vignette randomized across the whole sample(s) to rule out any potential of treatment cluster-based artefacts. Yet, researchers following this alternative approach should be aware that they would have to raise substantially larger samples to achieve the same level of power, which – due to increasing between-subject heterogeneity – might also induce further noise into the data, while the expected benefit of circumventing marginally small and statistically insignificant cluster effects is limited. Research pragmatism, hence, suggests that replicating the current study in its original design and vignette distribution procedure (two out of three) would be most advisable.

**A.3 Correlations and Reliabilities**

|  | | **1.** | | **2.** | | **3.** | | **4.** | | **5.** | | **6.** | | **7.** | | **8.** | | **9.** | | **10.** | | **11.** | | **12.** | | **13.** | | **14.** | |
| --- | --- | --- | --- | --- | --- | --- | --- | --- | --- | --- | --- | --- | --- | --- | --- | --- | --- | --- | --- | --- | --- | --- | --- | --- | --- | --- | --- | --- | --- |
| **Study variables** | |  |  |  |  |  |  |  |  |  |  |  |  |  |  |  |  |  |  |  |  |  |  |  |  |  |  |  |  |
| 1. | BRIBE | – |  |  |  |  |  |  |  |  |  |  |  |  |  |  |  |  |  |  |  |  |  |  |  |  |  |  |  |
| 2. | White treatment | .52 | *** | – |  |  |  |  |  |  |  |  |  |  |  |  |  |  |  |  |  |  |  |  |  |  |  |  |  |
| 3. | Grey treatment | -.13 | *** | -.50 | *** | – |  |  |  |  |  |  |  |  |  |  |  |  |  |  |  |  |  |  |  |  |  |  |  |
| 4. | Black treatment | -.39 | *** | -.50 | *** | -.50 | *** | – |  |  |  |  |  |  |  |  |  |  |  |  |  |  |  |  |  |  |  |  |  |
| 5. | High realism | .60 | *** | .50 | *** | -.17 | *** | -.32 | *** | – |  |  |  |  |  |  |  |  |  |  |  |  |  |  |  |  |  |  |  |
| 6. | Burnout | .04 |  | -.01 |  | -.00 |  | .01 |  | -.01 |  | – |  |  |  |  |  |  |  |  |  |  |  |  |  |  |  |  |  |
| 7. | CPI | -.09 | ** | .02 |  | -.03 |  | .01 |  | -.00 |  | -.01 |  | – |  |  |  |  |  |  |  |  |  |  |  |  |  |  |  |
| **Control variables** | |  |  |  |  |  |  |  |  |  |  |  |  |  |  |  |  |  |  |  |  |  |  |  |  |  |  |  |  |
| 8. | Risk propensity | -.06 |  | .00 |  | -.01 |  | .01 |  | -.09 | ** | -.02 |  | .07 | † | – |  |  |  |  |  |  |  |  |  |  |  |  |  |
| 9. | Female | -.09 | ** | .03 |  | -.06 | * | .03 |  | -.04 |  | .03 |  | .08 | ** | .02 | † | – |  |  |  |  |  |  |  |  |  |  |  |
| 10. | Age | .05 | † | -.03 |  | .04 |  | -.01 |  | -.03 |  | .00 |  | .07 | * | -.16 | *** | -.10 | *** | – |  |  |  |  |  |  |  |  |  |
| 11. | German | .05 | † | -.02 |  | -.03 |  | .00 |  | .08 | * | -.05 | † | .00 |  | -.41 | *** | .03 |  | .40 | *** | – |  |  |  |  |  |  |  |
| 12. | Belgian | -.06 | * | -.00 |  | -.00 |  | .00 |  | .00 |  | -.06 | * | .10 | *** | .47 | *** | -.01 |  | -.37 | *** | -.46 | *** | – |  |  |  |  |  |
| 13. | Dutch | -.04 |  | -.00 |  | .01 |  | -.01 |  | -.06 | * | .09 | * | -.09 | * | -.05 | † | -.03 |  | -.09 | ** | -.46 | *** | -.46 | *** | – |  |  |  |
| 14. | Community of faith | .02 |  | -.01 |  | -.01 |  | .01 |  | .01 |  | -.02 |  | .04 |  | -.10 | *** | .07 | * | -.08 | ** | .27 | *** | -.13 | *** | -.16 | *** | – |  |
| 15. | Study field | .02 |  | -.02 |  | .01 |  | .01 |  | .01 |  | -.02 |  | .08 | * | .07 | * | .02 |  | -.02 |  | .08 | ** | .26 | *** | -.35 | *** | .05 | † |

*Note*: Pooled data. † *p* < .10, * *p* < .05, ** *p* < .01, *** *p* < .001
